# Long-Term Phenotypic Evolution in GRIN2A-Related Disorders: Electroclinical and Genetic Insights from Two Families with Extended Follow-Up

**DOI:** 10.3390/genes16030323

**Published:** 2025-03-10

**Authors:** Ester Di Muro, Pietro Palumbo, Massimo Carella, Mario Benvenuto, Maria Rachele Bianchi, Umberto Costantino, Giovanni Di Maggio, Marco Castori, Giuseppe d’Orsi, Orazio Palumbo

**Affiliations:** 1 UOC Genetica Medica, Fondazione IRCCS Casa Sollievo della Sofferenza, 71013 San Giovanni Rotondo, FG, Italy; e.dimuro@operapadrepio.it (E.D.M.); p.palumbo@operapadrepio.it (P.P.); m.carella@operapadrepio.it (M.C.); m.benvenuto@operapadrepio.it (M.B.); m.castori@operapadrepio.it (M.C.); 2 Epilepsy Center—Neurological Unit, Fondazione IRCCS Casa Sollievo della Sofferenza, Viale Cappuccini, 71013 San Giovanni Rotondo, FG, Italy; mariarachelebianchi@gmail.com (M.R.B.); umbertocostantino94@gmail.com (U.C.); giov.dimaggio@gmail.com (G.D.M.)

**Keywords:** *GRIN2A*, targeted resequencing, neurodevelopmental disorders, epilepsy, cognitive impairment, behavioral disorders, electroencephalography, long-term follow-up

## Abstract

**Background:** The *GRIN2A* gene and its product protein have been linked to a wide spectrum of neurodevelopmental disorders named *GRIN2A*-related disorders. Clinical presentation is highly variable and characteristically includes acquired cognitive, behavioral, and language impairment, as well as epilepsy, ranging from benign forms to severe epileptic encephalopathy. Recent genetic investigations have expanded the clinical spectrum of heterozygous *GRIN2A* variants, improving our understanding of genotype–phenotype correlations. However, there have been few long-term observational studies of patients affected by the genetically determined *GRIN2A*-related disease. **Methods:** To understand the long-term changes in clinical features, we described three patients from two Italian families, carrying variants in the *GRIN2A* gene. **Results:** After more than a decade of extensive electro-clinical follow-up, we observed a progressive cognitive decline associated with severe behavioral disturbances, despite clinical seizure control. The persistent presence of EEG epileptiform abnormalities over time suggests the need for a longitudinal neurophysiological study to monitor disease progression and evaluate the potential for anti-seizure medication discontinuation. **Conclusions:** Our study offers new insights into the natural progression of epilepsy in *GRIN2A*-related disorders, highlighting that a more detailed understanding of the phenotype and timely, personalized treatment could enhance the management and quality of life for both *GRIN2A* patients and their caregivers.

## 1. Introduction

N-methyl-D-aspartate receptors (NMDARs) are ligand-gated ion channels, typically composed of two obligatory glycine-binding GluN1 subunits and two glutamate-binding GluN2 or GluN3 subunits, which are encoded by the *GRIN1*, *GRIN2A–D*, and *GRIN3A–B* receptor genes [1]. Each subtype of NMDA receptor exhibits distinct temporal and spatial expression patterns in the brain, localized in various cell types and subcellular compartments, contributing to a broad variety of protein functions [2]. These receptors play a critical role in mediating excitatory neurotransmission and are essential for neuronal development, synaptic plasticity, and fundamental cognitive processes such as learning, memory, and cognitive function.

*GRIN*-related encephalopathies represent a group of diseases characterized by epilepsy and intellectual disability associated with variants in one of the genes encoding one of the subunits of the NMDAR [3]. Between these, *GRIN2A*-related disease is the most common group of diseases, manifesting with acquired cognitive, behavioral, and language impairment, as well as various types of epilepsy, ranging from benign forms to severe epileptic encephalopathy with multi-drug resistance of which the long-term electro-clinical evolution has not been extensively studied.

From the phenotype point of view, variants in *GRIN2A* are predominantly associated with a more definite phenotype characterized by an epileptic spectrum ranging from Landau–Kleffner syndrome to benign childhood epilepsy with centrotemporal spikes, epileptic encephalopathy, atypical childhood epilepsy with centrotemporal spikes (ACECTS), and benign childhood epilepsy with centrotemporal spikes (BECTS) often associated with DD/ID [4,5]. In more than 80% of patients, *GRIN2A* variants cause epilepsy and speech disorders [6]. Furthermore, pharmacological manipulations that interfere with GluN2A have been associated with deficits in short-term memory, impaired fear memory formation, and compromised spatial working memory [7,8].

Here, we describe three adult patients with variants in *GRIN2A*, affected by developmental and epileptic encephalopathy, focusing on seizure semiology, EEG, and response to antiseizure medications (ASMs) to better delineate the electroclinical features, treatment options, and long-term outcome of epilepsy associated with *GRIN2A* variants.

## 2. Materials and Methods

### 2.1. Genomic DNA Extraction and Quantification

Peripheral blood samples have been taken from both the proband and his parents. The DNA extraction from peripheral blood lymphocytes and the relative quantitative dosage were obtained as previously described [9].

The family provided written informed consent for the molecular testing and the full content of this paper, and the study was performed according to the 1984 Declaration of Helsinki. The conservation and use of biological samples for scientific purposes were approved.

### 2.2. Next-Generation Sequencing Analysis

Proband DNA was analyzed by Targeted resequencing (TRS) by using a SureSelect gene panel (Agilent Technologies, Boulder, CO, USA) designed to selectively capture 135 known genes associated with genetic epilepsy.

Libraries preparation and NGS data analysis were performed as previously described [9].

The clinical significance of the identified putative variants was interpreted according to the American College of Medical Genetics and Genomics (ACMG) guidelines [10]. Variant analysis was performed taking the patient’s ethnicity into account. The variants were submitted to the LOVD database, and the nucleotide variant nomenclature follows the format recommended by the Human Genome Variation Society (HGVS, http://www.hgvs.org) (accessed on 25 July 2023).

### 2.3. Electro-Clinical Study

We conducted a comprehensive clinical and electroencephalographic (EEG) study on three patients from two unrelated families in the Apulia region of Southern Italy. These patients presented with developmental and epileptic encephalopathy due to *GRIN2A* gene mutations.

Data from each patient were collected, including demographic details, comprehensive epilepsy characteristics, the presence of additional symptoms, and the progression of epilepsy and other symptoms over time.

Additionally, all patients were administered the Wechsler Intelligence Scale for Children-Revised (WISC-R). EEG electrodes were placed according to the 10–20 International System with a bipolar montage. Signals were digitally acquired with a sampling frequency of 512 Hz and band-pass filters of 1.6–210 Hz, using equipment from Nihon Kohden (Tokyo, Japan).

## 3. Results

### 3.1. Electro-Clinical Features (See Table 1)

#### 3.1.1. Family 1

Patients 1 and 2 are siblings from non-related parents. Their father, carrying the c.1783del, p.(His595Metfs*59) variant, is asymptomatic.

**Table 1:** Clinical and instrumental features. y: years; ID: intellectual disability; FT: frontotemporal; SW: spike and wave; VPA: valproate; LEV: levetiracetam; CBZ; carbamazepine; ASM: anti-seizure medication.

	Patient 1	Patient 2	Patient 3
Sex	M	M	M
Age	25y	19y	24y
Age of Genetic Diagnosis	22y	17y	23y
ID	Severe	Severe	Severe
Speech delay	Yes	Yes	Yes
Behavior disorder	Yes	Yes	Yes
EEG	R FT and diffuse SW, disclosed by sleep	Normal	Normal
Seizure type	Tonic-Clonic in sleep	Tonic-Clonic in sleep	Tonic-clonic in wakefulness and sleep
Current ASM	VPA 1000 mg/day	VPA 500 mg/day	VPA 600 mg/day
Precedent ASMs	LEV, CBZ	CBZ	CBZ
Brain MRI	Normal	Normal	Normal

**Figure 1:**
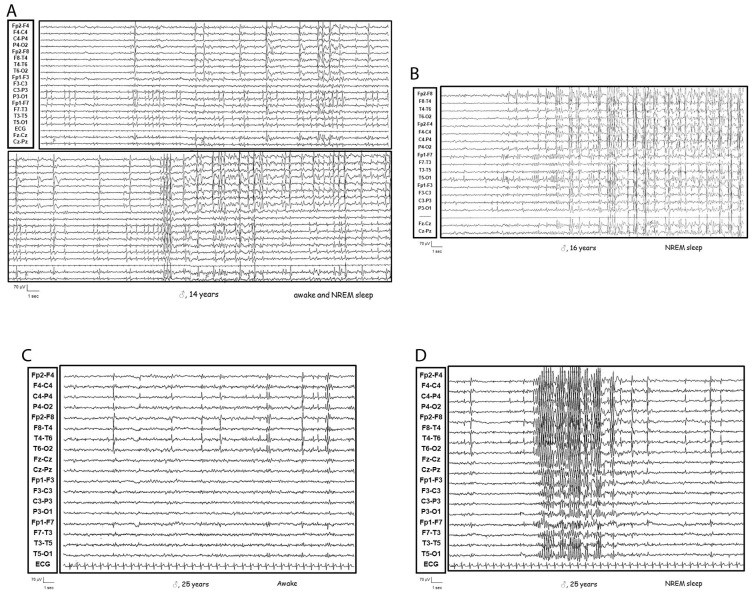
Patient 1 at (**A**) 14 years old: Asynchronous epileptiform abnormalities on bilateral fronto-centro-temporal derivations, markedly accentuated by sleep; (**B**) 16 years old: Asynchronous epileptiform discharges over bilateral fronto-centro-temporal regions, markedly accentuated during sleep, suggestive of a continuous spike-wave pattern, (**C**) 25 years old: epileptiform discharges over right fronto-centro-temporal region during awake; (**D**) 25 years old: epileptiform discharges over right fronto-centro-temporal region, markedly accentuated by sleep and with diffusion.

Patient 1 was a 25-year-old male born prematurely due to placental abruption whose early infancy was marked by language impairment, attention difficulties, and hyperactivity. At age 5, he began experiencing monthly focal motor seizures during sleep. EEG recordings revealed diffuse, asynchronous spike-wave and polyspike-wave discharges, particularly during non-REM sleep, with persistent epileptiform abnormalities in the right frontotemporal regions (see Figure 1). Despite polytherapy with valproate, levetiracetam, carbamazepine, and pulse steroids, seizures persisted. From age 10, he experienced cognitive and language decline, behavioral disturbances, and gradual seizure control.

**Figure 2:**
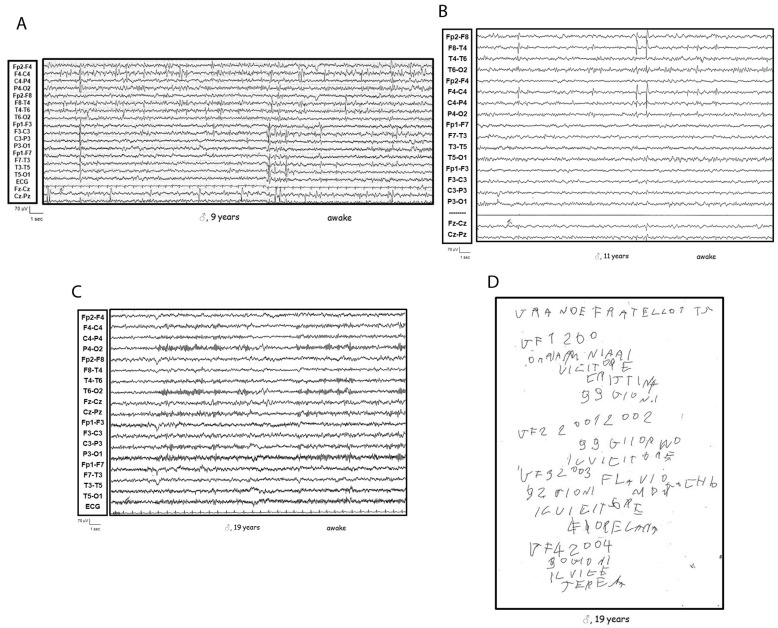
Patient 2 at (**A**) 9 years old: asynchronous epileptiform discharges over fronto-centro-temporal regions, accentuated by resting wakefulness; (**B**) 11 years old: epileptiform discharges over right fronto-centro-temporal region, accentuated by resting wakefulness; (**C**) 19 years old: EEG normal during resting wakefulness; (**D**) 19 years old: dysgraphia with an inability to construct sentences, suggesting a more severe underlying language disorder.

Patient 2 was a 19-year-old male who developed language impairment at age 2. EEG recordings revealed right fronto-temporal spike-wave and polyspike-wave discharges, predominantly during sleep. Over time, he experienced severe communication impairment, loss of reading and writing skills, and behavioral disturbances. At age 10, he had three focal motor seizures, which were controlled with valproate. In recent years, he has exhibited only behavioral disturbances, with normal awake EEG (see Figure 2).

#### 3.1.2. Family 2

Patient 3 is a 24-year-old male whose mother carries the c.2453C > T, p.(Ala818Val) variant and is asymptomatic. From infancy, he presented with severe speech and language impairments, as well as behavioral disturbances. At 13 months, he experienced febrile seizures, followed by non-febrile seizures during sleep at age 6. Valproate effectively controlled seizures. Longitudinal EEGs during wakefulness were normal.

### 3.2. Genetic Analysis

**Figure 3:**
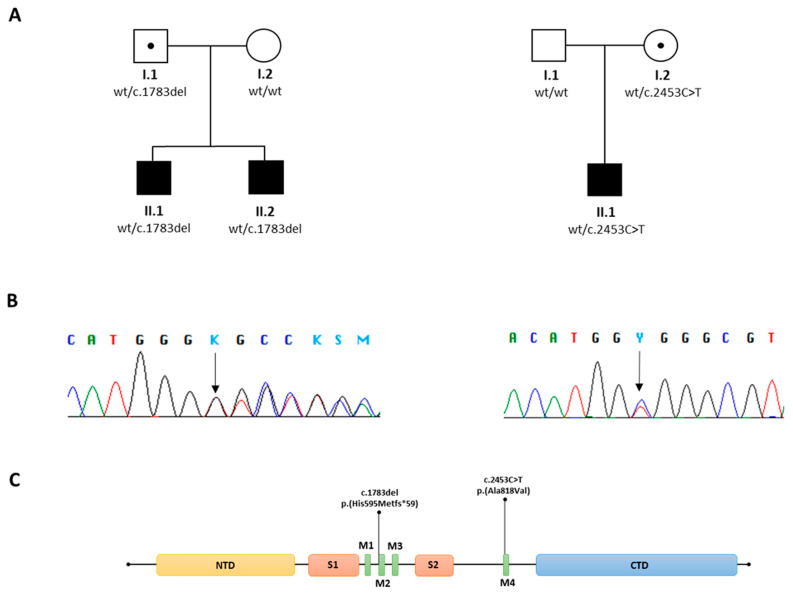
(**A**) Family pedigree with the phenotypes and segregation of *GRIN2A* mutations. (**B**) DNA sequence chromatogram of the *GRIN2A* mutations. Arrows indicate the positions of the mutations. (**C**) Schematic representation of *GRIN2A* protein and variants. Abbreviations: NTD, N-terminal domain; S1 and S2 segments form the glutamate-binding domain; M1–M4 transmembrane segments form the ion channel pore; CTD, C-terminal domain.

TRS enabled the identification of a novel variant, c.1783del, p.(His595Metfs*59), in exon 10 and c.2453C > T, p.(Ala818Val), in exon 13 of the *GRIN2A* gene (NM_000833). Both variants were detected with a depth coverage greater than 150× and high-quality scores (Phred quality > 3000 and genotype quality = 99). The first frameshift variant led to a premature stop codon and was absent in GnomAD, dbSNP, EP6500, and our in-house controls. Co-segregation analysis was performed in the family. The second missense variant causes an amino acid change and was also absent in GnomAD, dbSNP, EP6500, and our in-house controls. Bioinformatics details are provided in Table 2. Primers were designed using Primer3.0 (http://bioinfo.ut.ee/primer3-0.4.0) (accessed on 25 February 2023), with the following sequences: *GRIN2A*, exon 10 (Forward: GGCAATCACAGGACACAACT; Reverse: GTGCATTCGAGTTGATGGATCT) and *GRIN2A*, exon 13 (Forward: GCTGTGAGTTGCACATCGTC; Reverse: GAGCATCCAGATTTTGTGCA). PCR was performed under standard conditions, and the PCR products (479 bp and 478 bp) were sequenced on an ABI 3500xL DNA Analyzer (Applied Biosystems, Foster City, CA, USA). Parental DNA analysis confirmed the variant as a de novo event (Figure 3). Based on ACMG guidelines, the detected variant was classified as likely pathogenic.

## 4. Discussion

*GRIN2A* mutations have previously been associated primarily with idiopathic focal epilepsy with incomplete penetrance [4,5,11] and, less frequently, with epileptic encephalopathy (EE) [12,13].

As far as we know, to date, approximately 250 individuals affected by *GRIN2A*-related disorders have been recorded showing a wide range of clinical manifestations [14]. Null variants, including microdeletions, and missense variants within the amino-terminal domain (ATD) and the ligand-binding domains (LBD) S1 and S2 cause a significant reduction or loss of NMDARs biological activity, leading to mild phenotypes [15]. In contrast, missense variants within the three transmembrane domains (TMDs) and linker domains predominantly lead to an increase in NMDAR activity, which correlates to more severe phenotypes [14,16]. The position of the variants in different functional domains suggests that their clinical effects might also differ. Therefore, electrophysiological analysis is a useful method to understand how a variant causes disease and, importantly, to evaluate possible treatment options.

Here, we described two families from the Apulia region, in Southern Italy, carrying a variant in the *GRIN2A* gene identified employing TRS, which was able to reveal two heterozygous *GRIN2A* variants, c.1783, p.(His595Metfs*59) in the first and c.2453C > T, p.(Ala818Val) in the second family, respectively. The variant c.1783del, p.(His595Metfs*59) is of paternal origin while the variant c.2453C > T, p.(Ala818Val) is maternal. Both parents are unaffected carriers. Previous studies described the incomplete penetrance and variable expressivity of *GRIN2A* mutations [5,11]. This scenario was also observed in the families described here but the underlying mechanism remains poorly understood. The numerous genomic variations in each individual and environmental factors might modify the phenotype. However, previous research has shown that the locations of missense mutations influenced the severity of developmental phenotypes, suggesting that the traits observed in the patients could reasonably be affected by a sub-regional molecular effect of *GRIN2A* mutations. Furthermore, in our patients, we cannot exclude potential founder effects or other population-specific factors able to mediate and/or influence both the severity and the variability of the clinical traits found since they came from a specific region in Southern Italy (Apulia). To understand the functional impact of the identified variants and to shed light on some molecular aspects that are currently unknown and could help explain the incomplete penetrance and variable expressivity already known and widely documented in *GRIN2A*-related disorders, functional studies and/or animal models are needed.

Although *GRIN2A*-related disorders have been well characterized from a phenotypic standpoint and the genotype–phenotype correlations have been widely documented, the long-term electro-clinical evolution has not been extensively studied. The present patients underwent extensive clinical and instrumental follow-up (>10 years). We observed a clinical picture characterized by cognitive decline and behavioral disturbance despite the complete control of epilepsy seizures, generalized tonic–clonic or focal motor that started in early childhood and especially during sleep were rapidly controlled with monotherapy, and, at the time of the last clinical evaluation, all patients have been seizure-free for several years.

Serial EEGs over the years showed epileptiform abnormalities prevalent in the temporal regions, disclosed and marked by sleep and often continuous, suggesting an electro-clinical pattern similar to those with Landau–Kleffner syndrome [17]. Interestingly, in one patient, we also observed the persistence of this electro-clinical pattern during sleep in adulthood.

In conclusion, a long-term electro-clinical follow-up in three patients with *GRIN2A* mutations suggests a clinical picture characterized overall by severe cognitive and behavioral disturbances. In addition, although rapid and complete control of seizures is reached, the persistence of epileptiform abnormalities during sleep suggests longitudinal neurophysiological studies in order to detect the monitoring of the disease progression and to provide an eventual suspension of ASMs.

In this regard, our study provides insights useful to suggest that a multidisciplinary team with neurologists, psychiatrists, and psychologists is mandatory in order to organize the appropriate management and give support to parents and caregivers.

## Figures and Tables

**Table 2:** Bioinformatic details of variants.

Chromosome	Position	Reference Allele	Alternative Allele	Genotype	Gene	Nucleotide Change	Amminoacid Change	dbSNP ID	TOPMED Allele Count	GnomAD ALL Allele Count
16	9923502	GG	G	Het	*GRIN2A* (NM_000833)	c.1783	c.1783del, p.(His595Metfs*59)	rs779219028	N.A.	0.00000398
16	9862850	C	T	Het	*GRIN2A* (NM_000833)	c.2453C > T	p.(Ala818Val)	rs751455326	N.A.	0.00000398

## Data Availability

The data presented in this study are reported in the Leiden Open Variation Databases (LOVDs) (https://databases.lovd.nl/shared/variants/0000933303#00001108; https://databases.lovd.nl/shared/variants/0000933302#00001108) (accessed on 6 September 2023).
